# 

**Published:** 1859-03

**Authors:** 


☞ At a meeting of the class of '59 of the Medical Department of the University of Buffalo, N. L. Bates was called to the chair and H. R. Stagg was appointed secretary.

The following resolutions were unanimously adopted:

*Resolved*, That we tender our warmest and most sincere thanks to Dr. Austin Flint, Jr., for his lectures on Physiology, and we congratulate him on his successful *debut* as a teacher in this important department of medical education.

*Resolved*, That we consider him worthy of our hearty commendations, inasmuch as he has been one of the first men in this country to illustrate his lectures by demonstrations upon the living subject.

*Resolved*, That we, as a class, advise all medical students to listen to this most able lecturer in the department of his choice, assuring them that all will find in him the scholar, the gentleman, and friend.

*Resolved*, That a committee of five be appointed to present the above resolutions to the Buffalo Medical Journal and to the daily papers of our city for publication.

The following gentlemen were appointed such committee in accordance with the above resolutions:—C. C. Dellenbaugh, F. M. Byington, W. W. Potter, L. Damainville, U. C. Lynde.

Dalton's Treatise on Human Physiology, may be obtained of Mr. A. I. Mathews, by mail, postage paid. Price $4.25.

